# The effects of aging on neuropil structure in mouse somatosensory cortex—A 3D electron microscopy analysis of layer 1

**DOI:** 10.1371/journal.pone.0198131

**Published:** 2018-07-02

**Authors:** Corrado Calì, Marta Wawrzyniak, Carlos Becker, Bohumil Maco, Marco Cantoni, Anne Jorstad, Biagio Nigro, Federico Grillo, Vincenzo De Paola, Pascal Fua, Graham William Knott

**Affiliations:** 1 BioEM Facility, School of Life Sciences, Ecole Polytechnique Fédérale de Lausanne, Lausanne, Switzerland; 2 Biological and Environmental Sciences and Engineering, King Abdullah University of Science and Technology, Thuwal, Saudi Arabia; 3 BioEPIX, XLI M, Faculty of Science and Technology, University of Limoges, Limoges, France; 4 Computer Vision Laboratory, Ecole Polytechnique Fédérale de Lausanne, Lausanne, Switzerland; 5 Centre for Electron Microscopy, Ecole Polytechnique Fédérale de Lausanne, Lausanne, Switzerland; 6 MRC Centre for Developmental Neurobiology, King's College London, New Hunt's House Guy's Campus, London, United Kingdom; 7 MRC Clinical Science Centre, Faculty of Medicine, Imperial College London, London, United Kingdom; Nathan S Kline Institute, UNITED STATES

## Abstract

This study has used dense reconstructions from serial EM images to compare the neuropil ultrastructure and connectivity of aged and adult mice. The analysis used models of axons, dendrites, and their synaptic connections, reconstructed from volumes of neuropil imaged in layer 1 of the somatosensory cortex. This shows the changes to neuropil structure that accompany a general loss of synapses in a well-defined brain region. The loss of excitatory synapses was balanced by an increase in their size such that the total amount of synaptic surface, per unit length of axon, and per unit volume of neuropil, stayed the same. There was also a greater reduction of inhibitory synapses than excitatory, particularly those found on dendritic spines, resulting in an increase in the excitatory/inhibitory balance. The close correlations, that exist in young and adult neurons, between spine volume, bouton volume, synaptic size, and docked vesicle numbers are all preserved during aging. These comparisons display features that indicate a reduced plasticity of cortical circuits, with fewer, more transient, connections, but nevertheless an enhancement of the remaining connectivity that compensates for a generalized synapse loss.

## Introduction

Aging is associated with a decline of faculties such as memory, motor skills, and sensory perception. These deficits, however, are not thought to be due to a substantial loss of neurons, but rather subtler changes at the level of connectivity, neuronal morphology and white matter structure [1, 2, 3, 4]. However, although many structural studies have investigated how neuronal connectivity may be altered in the aging brain, these have typically focussed on one particular feature such as the synapse, or specific classes of dendrite, or axon. It is unclear, at the ultrastructural level, how the architecture of the brain’s neuropil, in any single region, may be altered during aging, or indeed to what extent changes to the morphology of dendrites and axons manifest as alterations in synaptic connectivity. Electron microscopy analyses have typically counted the number of synapses in defined volumes. These have shown that synapse loss is present in various brain regions, for example in the monkey prefrontal and visual cortex [5, 6, 7]. However, though these measurements give an indication of connectivity, their value can be misleading. As the amount of dendrite or axon in the sampled volumes may change with age [6, 8], considering only the numbers of synaptic connections without the total lengths of neurites present may not be a true indication of alterations in their degree of connectivity.

Age-related changes to axon and dendrite morphology have been shown using light microscopy of different cell types in various brain regions. The impact on their connectivity, however, can only be alluded to the basis of correlations between the dendritic spine and axonal bouton volumes, and synapse size [9, 10, 11]. Lower spine densities are seen on dendrites in the visual cortex, prefrontal cortex, and the hippocampus [12, 13, 14, 15, 16, 17]. In the somatosensory cortex, however, a detailed longitudinal analysis of spines on the apical dendrites of layer V pyramidal neurons showed no loss, but rather a decrease in spine volume [18]. In contrast, an analysis of spine size in the prefrontal cortex showed a spine volume increase, that accompanied the spine loss; principally due to the removal of only thin spines [13, 15, 19].

While dendrites and their spines have received much attention, age-related changes to axons have not. A decrease in synatophysin immuno-reactivity in the hippocampus, for example, only indicates a reduction in the synaptic bouton machinery [20], but in the cortex the live imaging of aged axons showed no change in bouton density, but an increase in their size [18, 21]. As functional analysis has suggested that a disturbance of inhibitory balance is a feature of the aging sensory cortex, with a decrease being shown in visual and auditory cortices in monkeys and rodents [22, 23] this raises questions as to the effect of aging on the different synapse populations.

The current study, therefore, has examined neuropil ultrastructure with analyses of not only the synapse densities and synapse size but also all the axons and dendrites contained in discrete volumes. This included a size analysis of all dendritic spines and all axonal boutons. This allowed us to understand how the neuropil as a whole, was affected by aging, not just the concentrations of synaptic connections.

We used focussed ion beam scanning electron microscopy [24] (FIBSEM) at a suitable resolution so that semi-automated segmentation methods could be used. Within the imaged volumes, all synapses were classified as excitatory or inhibitory, based on their morphology. Their sizes were also measured. Within sub-volumes of each image stack, dense reconstructions were made of all the enclosed axons and dendrites, as well as their mitochondria.

We imaged layer 1 of the somatosensory cortex. This is a region that has been used in many studies of neural plasticity. Layer 1 in particular has been the site of a number of *in vivo* imaging analyses of dendritic and axonal structure—during development, adulthood, and also aging [25, 26, 18, 21]. The analysis was confined to the middle of this layer and gives a comprehensive picture of its architecture, the effect of aging, and how the connectivity is altered. Despite a synapse loss, there were proportionally less inhibitory connections, resulting in an increase in the excitatory/inhibitory balance. The remaining excitatory synapses were also larger so that total synapse surface area within the volume, or along the axons and dendrites, remained the same. The correlations between the sizes of synapses, spines, boutons, and vesicle populations were also similarly correlated in the aged neuropil, as in the adult. These analyses show the subtle structural alterations of aging neuropil that accompany synapse loss, indicating a connectivity that is less plastic, yet with all the hallmarks of morphology that has maintained a strong excitatory drive.

## Materials and methods

### Perfusion and sample preparation

The animals used in this study were bred and housed in the animal facility of the MRC Clinical science centre (Imperial College, London, UK) according to local regulations. Mice were handled and sacrificed by V de Paola according to the regulations of the approved UK project license (V de Paola 70/7845) and in accordance with the Animals (Scientific Procedures) Act 1986 (United Kingdom) and associated guidelines released by the Animals in Science Committee. No animal showed any signs of suffering or distress during anaesthesia.

Six male C57BL6 mice (N = 3 were 4 months old, N = 3 were 24 months old) were euthanized with a mix of ketamine and zylazine, and then immediately perfused via the heart with 2.5% glutaraldehyde and 2% paraformaldehyde in 100 mM phosphate buffer, pH 7.4. The fixed brains were sliced at a thickness of 60 micrometers and sections, including the barrel cortex, were selected for further processing. The tissue was washed in cacodylate buffer (0.1 M, pH 7.4) and post-fixed for 40 min with 1.5% potassium ferrocyanide and 1% osmium tetroxide, again for 40 min in 1% osmium tetroxide alone, each in 0.1 M cacodylate buffer, and finally for 40 min in 1% aqueous uranyl acetate. Sections were then dehydrated in a graded alcohol series, infiltrated with Durcupan resin overnight, and finally embedded in fresh resin between glass slides. The resin was hardened at 65° C for 24 hours and then the embedded section glued to a flat, 1-millimeter thick slab of blank resin for trimming in the ultramicrotome.

### Light microscopy

**Fig 1:**
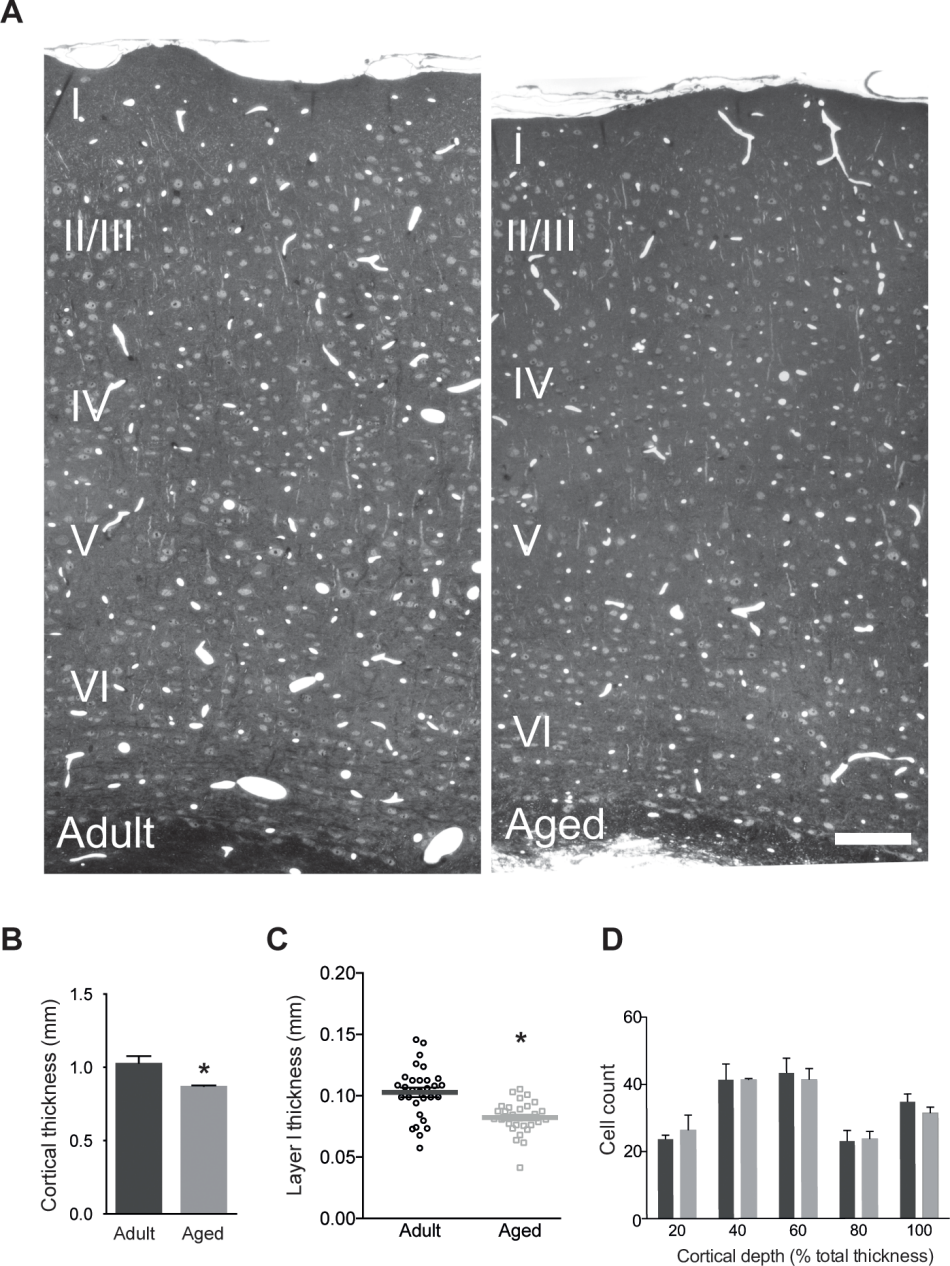
Somatosensory cortex of aged mice is thinner than adults. **A**, Semi-thin coronal sections of mouse somatosensory cortex indicating a reduced cortical thickness in the aged example (left, adult, 4 months old; right, aged, 24 months old). **B**, Measurements of cortical thickness from 3 aged and 3 adult mice, in the semi-thin sections, showed a reduction of 15.6%, (adult, 1.03 ± 0.047 mm; aged, 0.87 ± 0.003 mm; unpaired t-test, p = 0.0297). **C,** Measurements of layer I thickness from the same mice, in the semi-thin sections, showed a reduction of 19.4% (adult, 0.103 ± 0.004 mm; aged, 0.083 ± 0.002 mm; N = 10 per each of the six mice; p = 0.0009, unpaired t-test). **D**, Counts of cell profiles from sections used in **B**, across the cortical thickness, show no difference between adult and aged animals in any of the five bins positioned from the pial surface to the white matter. Bars indicate the mean ± sem. Differences are not statistically significant; Kolmogorov-Smirnov test, p > 0.9. Scale bar in **A** is 100 micrometers.

From each of the six animals, 1 micrometer-thick sections, including all the cortical layers from the pial surface to the white matter, were cut from somatosensory cortex using an ultramicrotome (UC6, Leica Microsystems). We selected sections in which we could identify the large cellular arrangements in layer IV, known as barrels, corresponding to the animals’ whiskers. Sections were stained with toluidine blue for 10 minutes on a hot plate at 60 degrees and then rinsed twice before imaging with a light microscope (Zeiss, AxioSkop2) at 20 times magnification (**Fig 1A**). We acquired 6 images per section, through the entire cortical thickness, then stitched them together using Photoshop software (Adobe). Total cortical thickness was measured as the distance between the pial surface, above layer 1, and the bottom of layer 6, at the point where the white matter starts (**Fig 1B**). Layer I thickness was measured as the distance between the pial surface and the bottom of layer I, at 10 different positions along the pial surface per sample (**Fig 1C**). The total thickness was then divided into 5 equal sized bins, and cell profiles counted, but only when the nucleoli were visible. The total number of cells, per bin, was averaged between animals in each group (**Fig 1D**).

### Imaging

The block used to produce the semi-thin section through the cortical thickness was stuck to an aluminium stub, with conductive carbon paste, and coated with a 10 nm layer of gold (Cressington vacuum evaporation system). Serial images were collected using a focussed ion beam scanning electron microscopy (FIBSEM; NVision 40, Zeiss NTS, Oberkochen, Germany) as described previously [27]. For the final serial imaging, we used an acceleration voltage of 1.8 kV with a current of between 340 and 400 pA, and dwell time of 10 μs per pixel. The image stacks were acquired in the middle of layer 1 in each of the animals. The final stacks were aligned using the MultiStackReg addon [28] available for the FIJI image processing software (www.fiji.sc). Each one of the volumes from the six mice was between 660 and 1520 μm^3^ (four-month-old mice; 849.7 μm^3^, 1520.7 μm^3^, 705.6 μm^3^ 24-month-old mice; 1416.7 μm^3^, 915.8 μm^3^, 662.6 μm^3^).

### Synapse quantification

**Fig 2:**
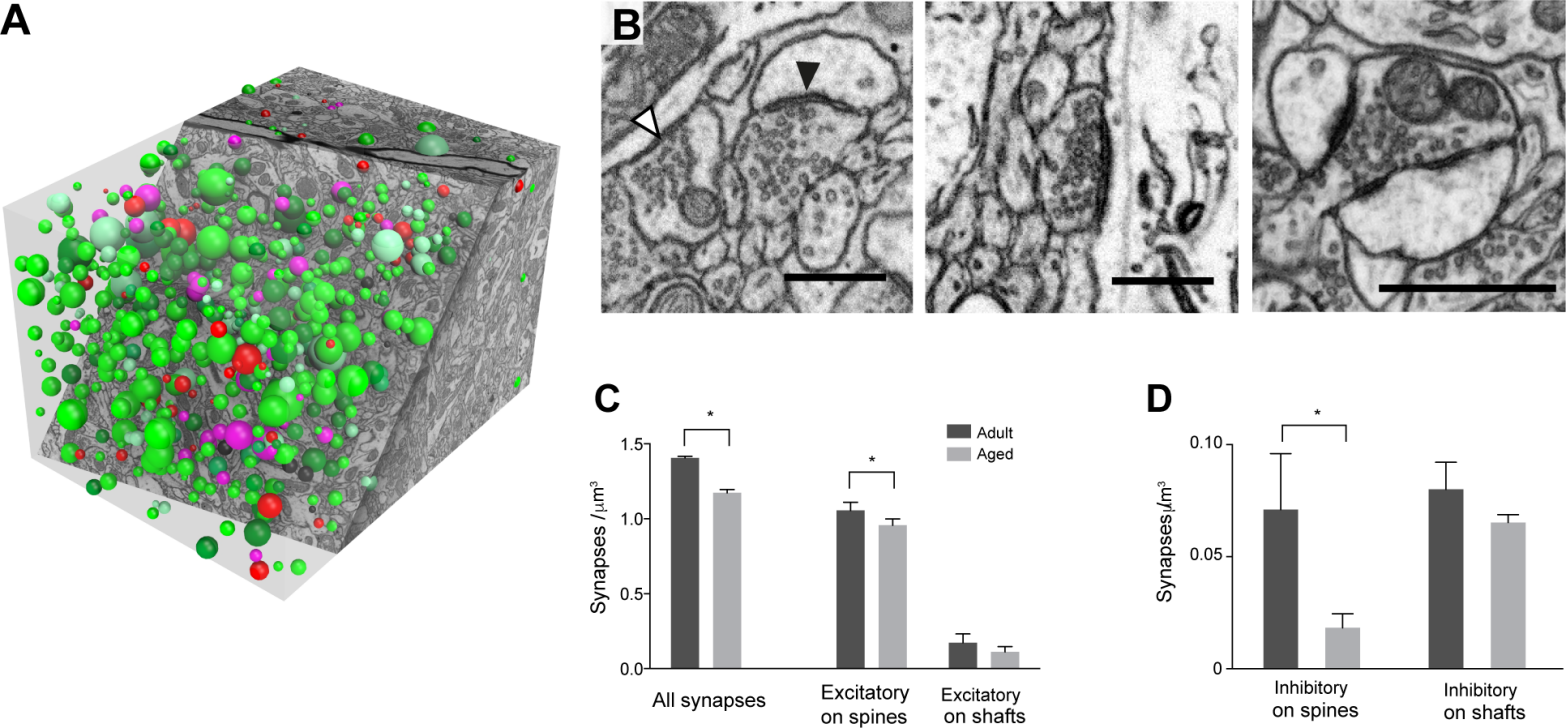
Synapse density decreases per unit volume in adult and aged mouse layer 1 neuropil. **A**, To count synapses in stacks of serial FIBSEM images (3 adult, and 3 aged), circles were placed at the center of each synapse using the TrakEM2 software in FIJI (www.fiji.sc); with their diameter equal to the maximum diameter of each contact. **B**, micrographs showing: left, an asymmetric synapse on a spine (presumed glutamatergic, black arrowhead) and a symmetric synapse on a shaft (presumed inhibitory, white arrowhead); center, an asymmetric synapse on a shaft; right, a multi-synaptic bouton (MSB). **C,** The density of all synapses was significantly lower in aged mice (adult, 1.40 ± 0.006 per μm^3^; aged, 1.17 ± 0.013 per μm^3^; t-test, *, p < 0.001) with a significant drop in asymmetric synapses on dendritic spines (adult, 1.05 ± 0.03 per μm^3^; aged, 0.96 ± 0.02 per μm^3^; t-test, *, p < 0.001). On dendritic shafts, there were less asymmetric synapses, but the drop was not significant (adult, 0.17 ± 0.03 per μm^3^; aged, 0.11 ± 0.02 perμm^3^; t-test, p = 0.09). **D**, Symmetric (presumed inhibitory) synapses were also significantly reduced, but only on dendritic spines (adult, 0.07 ± 0.01 per μm^3^; aged, 0.02 ± 0.006 per μm^3^; t-test, *, p = 0.0028) and not on shafts (adult, 0.08 ± 0.07 per μm^3^; aged, 0.065 ± 0.003 per μm^3^; t-test, p = 0.27). Scale bar in **B** is 500 nanometers.

Synapse size and densities were measured in these complete imaged volumes (**Fig 2**) that totaled 6,071 cubic micrometers (**Table 1**). They were classified according to their morphology (**Fig 2B**) as either asymmetric (presumed glutamatergic) or symmetric (presumed GABAergic). We also categorized the postsynaptic element as either dendritic shaft or spine. By placing the counting frame one micrometer inside the edge of the image, we were able to clearly identify structures beyond the square in which they were plotted. All synapses were classified using two independent observers who reached consensus on the identities of the different structures. Synaptic densities were calculated by using inclusion planes on three sides of the volume (left, top and upper) and exclusion planes on the other three (right, bottom and lower). With this method, it was possible to classify 98.9% of all synapses. It must be noted, however, that although the majority of synapses in the cortex are glutamatergic (asymmetric) and GABAergic (symmetric), we cannot discount that a small percentage of other classes, namely neuromodulatory, may have been included incorrectly.

**Table 1:** Synapse density per type.

Animal	Age	total vol.	No. Synapses	Total density	Asymmetric on spines single bouton	Asymmetric on spines multisynaptic bouton	Asymmetric on dendrite single bouton	Asymmetric on dendrite multisynaptic bouton	Symmetric on spine	Symmetric on dendrites	Unknown
**1**	4 months	849,7224	1190	1,400	0,920	0,080	0,221	0,020	0,073	0,072	0,014
**2**	24 months	1416,69	1627	1,148	0,805	0,132	0,078	0,001	0,030	0,059	0,021
**3**	4 months	1520,7	2158	1,419	0,882	0,172	0,116	0,022	0,095	0,074	0,015
**4**	24 months	915,84	1080	1,179	0,850	0,080	0,142	0,008	0,016	0,066	0,016
**5**	24 months	662,592	790	1,192	0,838	0,168	0,103	0,003	0,009	0,071	0,002
**6**	4 months	705,6	989	1,402	0,959	0,152	0,113	0,027	0,045	0,094	0,011

Measurements of synapse density per each type on each fully imaged volume.

**Fig 3:**
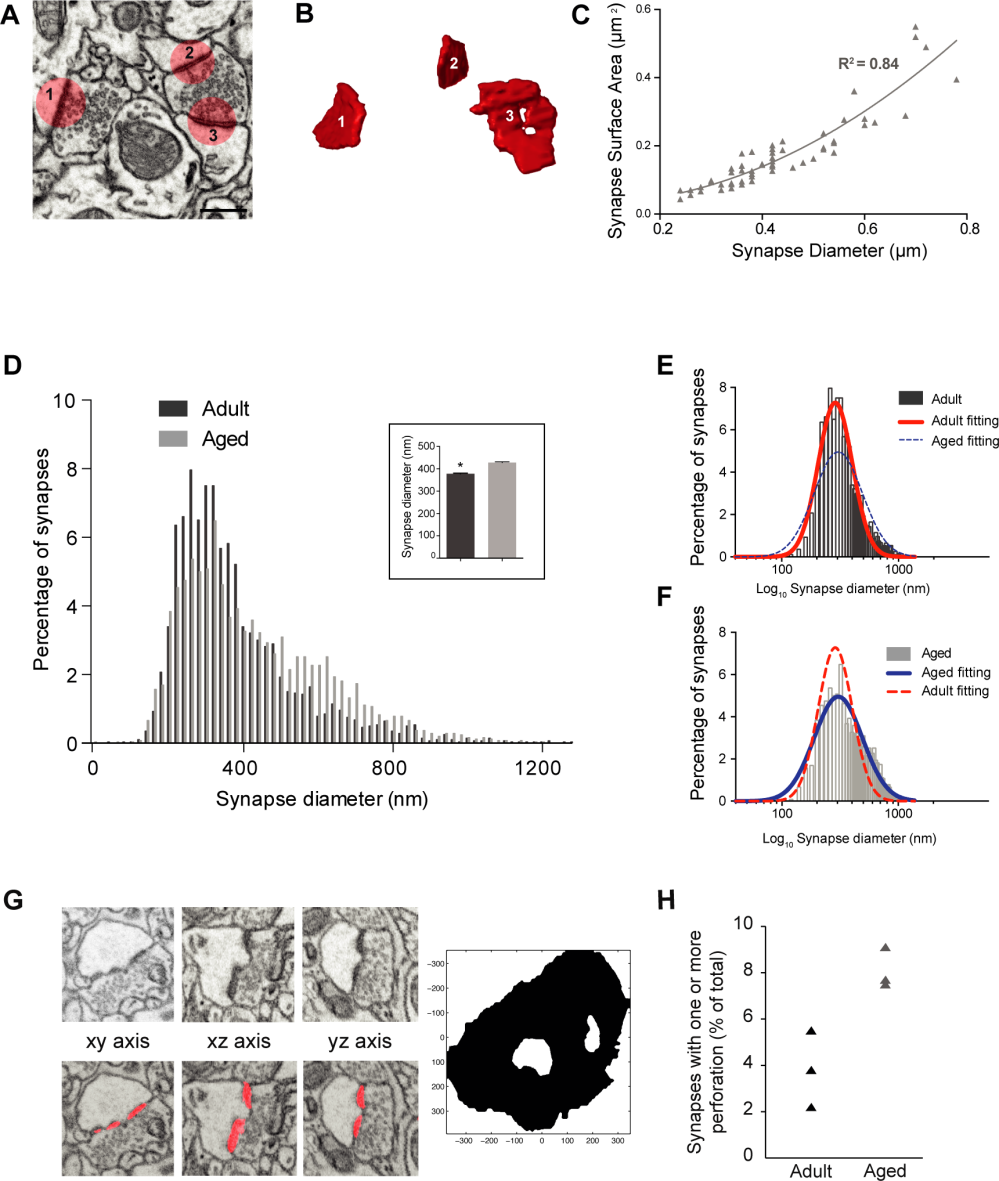
Synapses are larger in aged layer 1 neuropil. **A**, FIBSEM electron micrograph shows two boutons making asymmetric synapses; synapse 1 is made with a single synaptic bouton, 2 and 3 with a multi-synaptic bouton. **B**, the same synapses were segmented in the TrakEM2 software in FIJI (shown in red: www.fiji.sc) and reconstructed in 3D. **C**, There is a strong correlation between the diameter of circles used to annotate 57 synapses and their surface areas measured from their 3D reconstruction (slope of second polynomial regression, y = 0.032–0.09x + 0.95x^2^; R^2^ = 0.84). **D,** Frequency distribution of asymmetric synapse sizes, estimated from the maximum diameter measurements shown in **Fig 2** (Inset shows average values of all measurements, N = 2800 adult synapses, 377.8 ± 3.2 nm; N = 2423 aged synapses, 427.7 ± 3.9 nm; p < 0.0001, Kolmogorov-Smirnov test), showed that synapses in layer 1 of aged mice are larger. **E**, The same data plotted on a logarithmic scale for adult, and **F**, aged mice. Both show a log-normal distribution (adult, red fitting; center = 287.5, amplitude = 7.27, width = 0.33, R^2^ = 0.96; aged, blue fitting; center = 305.1, amplitude = 4.9, width = 0.47, R^2^ = 0.94). **G**, Examples of a series of micrographs containing a perforated synaptic density (top) and its segmentation (bottom) by means of the automated detection method, and its 3D rendering on the right. **H**, Graph showing the percentage of asymmetric synapses that displayed one or more perforation.

The synapses were labeled in the stack of serial images using the TrakEM2 software operating in FIJI [29], by placing a ball object at the center of the synaptic contact, whose diameter matched the maximum width of the synapse. This estimate of synapse size from the diameter of these balls correlates closely with their actual size measured from segmentations of synapses (**Fig 3C**; R^2^ = 0.84). Details of synapse reconstruction and measurements are given below.

### Analysis of neuronal morphology

**Fig 4:**
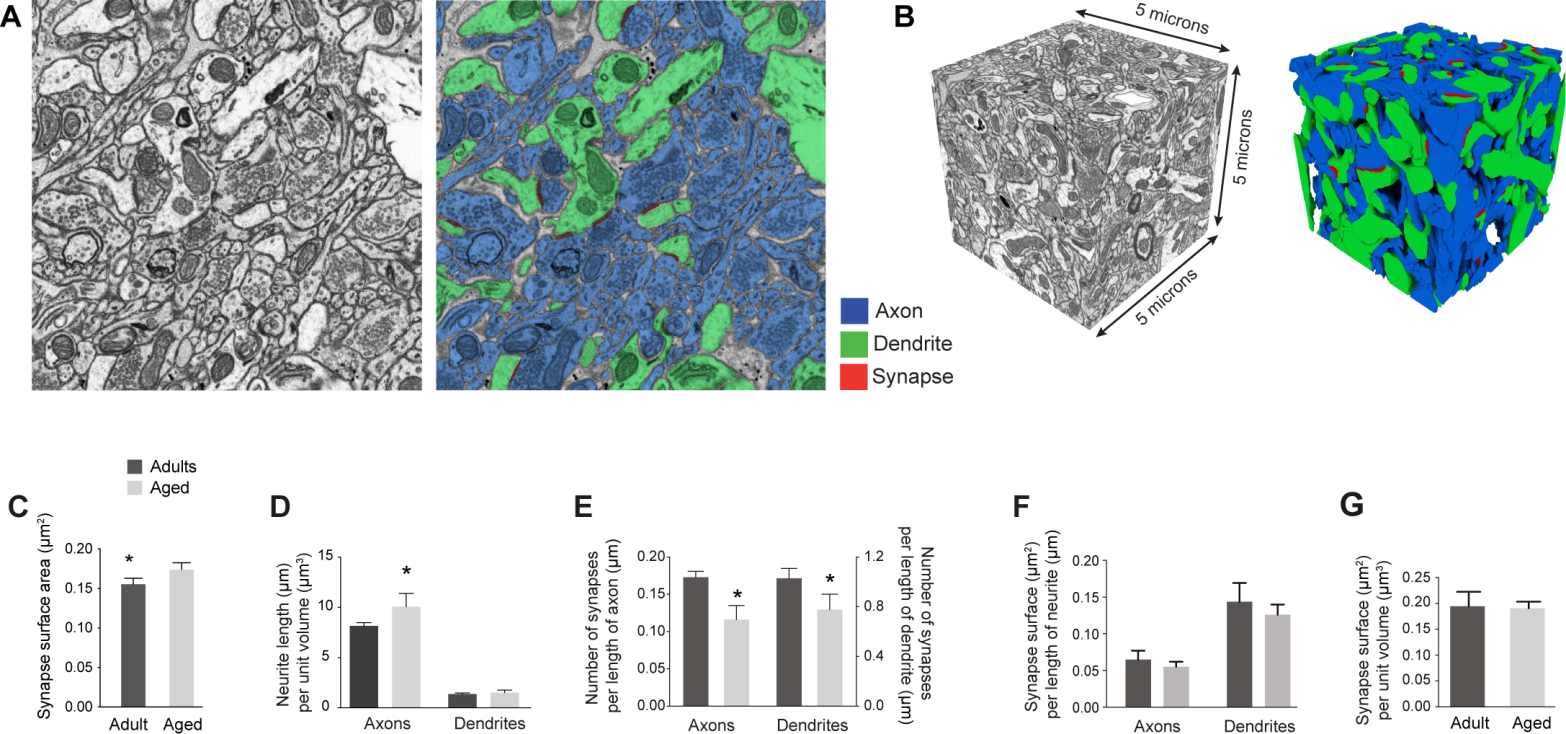
Dense reconstructions of sub-volumes reveal the same amount of asymmetric synaptic surface area per unit length of axon and dendrite, and per unit volume of neuropil in adult and aged mice. **A**, FIBSEM images were segmented in the Ilastik software (www.ilastik.org) to reconstruct all the axons (shown in blue) and dendrites (in green). Synapses were segmented in the TrakEM2 software in FIJI (shown in red: www.fiji.sc). **B**, These stacks were sub-volumes from the larger ones used for synapse density measurements (Fig 2A) and had side lengths of 5μm (volumes of 25 μm^3^). All axons, dendrites and synapses were reconstructed in six of these cubes (3 adults, 3 aged). **C**, Average synaptic surface area shows a significant increase in aged animals (adult, N = 313 synapses 0.15 ± 0.007 μm^2^ per μm^3^; aged, N = 288 synapses 0.17 ± 0.008 μm^2^ per μm^3^; Kolmogorov-Smirnov test, p = 0.04). **D**, Analysis of neurite lengths shows that layer 1 neuropil of aged mice contained significantly more micrometers of axon per cubic micrometer of neuropil (8.15 ± 0.19 μm of axons per μm^3^ for adults; 10.27 ± 0.88 μm of axons per μm^3^ for aged; p = 0.016, unpaired t-test). The dendritic content was smaller but non-significant (1.37 ± 0.06 μm of dendrites per μm^3^ for adults; 1.53 ± 0.12 μm of dendrites per μm^3^ for aged; p = 0.3, unpaired t-test). **E**, Number of asymmetric synapses per unit length of axon was 32.9% less in aged mice (adult, 0.173 ± 0.005 synapses per μm of axons; aged, 0.116 ± 0.011 synapses per μm of axons; t-test, p = 0.008). Numbers of asymmetric synapses per unit length of dendrite was 24.6% less in aged mice (adult, 1.03 ± 0.046 synapses per μm of dendrite; aged, 0.776 ± 0.072 synapses per μm of dendrite; t-test, p = 0.04). **F**, Asymmetric synaptic surface area per unit length of axons or dendrites was not significantly different mice (adult, 0.065 ± 0.012 μm^2^ per μm of axons; aged, 0.055 ± 0.007 μm^2^ per μm of axons; unpaired t-test, p = 0.53; adult, 0.144 ± 0.026 μm^2^ per μm of dendrites; aged, 0.126 ± 0.014 μm^2^ per μm of dendrites; unpaired t-test, p = 0.57). **G**, Asymmetric synaptic surface per unit volume also showed no difference between the adult and aged mice (adult, 0.19 ± 0.03 μm^2^ per μm^3^; aged, 0.19 ± 0.012 μm^2^ per μm^3^; t-test, p = 0.9).

From each of the six volumes, a single sub-volume measuring 5 x 5 x 5 μm^3^ randomly chosen within each of the image stacks was selected for dense reconstruction of every neuronal element, including all the synapses and mitochondria (**Fig 4**). Axons and dendrites were reconstructed using the carving, semi-automated algorithm [30] of the Ilastik software 0.5 (www.ilastik.org). The synapses were reconstructed using the TrakEM2 software, by drawing the contact site between pre and post-synaptic densities as a single object. The same method was used for mitochondria. To enable rapid and accurate reconstructions on a desktop computer (MacPro, 2 x 2.4 GHz Quad-Core Intel Xeon, 12 GB 1066 MHz DDR3), image stacks were downsampled to half of their original size prior to importing them into Ilastik [31, 32]. From the six volumes, 1820 pieces of axon and 478 of dendrite were reconstructed (details in **Table 2**). A total of 1135 synaptic contacts were manually reconstructed.

**Table 2:** Measurements on saturated reconstructions.

	4 Months old			24 Months Old		
	1	3	6	2	4	5
**Axons**						
***Total***	1019,68	977,40	1059,31	1345,96	1069,19	1360,67
***Excitatory***	358,50	358,38	434,99	419,27	379,19	497,70
***Inhibitory***	133,31	96,28	90,02	116,48	89,45	83,05
***Unknown***	155,17	54,21	112,14	146,52	220,50	187,23
***non-connecting neurites***	372,69	468,52	422,16	663,70	380,06	592,70
**Dendrites**						
***Total***	165,63	162,64	186,42	197,55	215,38	153,75
**Boutons**			** **			
***Total***	235	236	199	205	210	205
*Complete*	142	157	114	101	95	86
*Incomplete*	93	79	85	104	115	119
*Total surface area (complete only)*	254,79	248,69	175,47	148,72	176,84	150,96
*Mean for each bouton*	1,79	1,58	1,54	1,47	1,86	1,76
*Standard deviation*	1,18	0,94	1,04	1,04	1,19	1,21
*Total volume (complete only)*	23,55	21,36	15,12	13,91	16,62	13,34
*Average*	0,17	0,14	0,13	0,13	0,17	0,16
*Standard deviation*	0,17	0,12	0,14	0,12	0,17	0,13
***Excitatory***	148	112	146	133	110	133
*Complete*	85	84	83	61	53	63
*Incomplete*	148	112	146	72	57	70
*Total surface area (complete only)*	162,86	145,71	137,69	87,95	120,38	118,95
*Mean for each bouton*	1,92	1,73	1,66	1,44	2,27	1,89
*Standard deviation*	1,15	0,93	1,15	0,86	1,28	1,17
*Total volume (complete only)*	14,88	12,89	12,41	8,73	12,51	11,39
*Average*	0,18	0,15	0,15	0,13	0,24	0,18
*Standard deviation*	0,14	0,12	0,16	0,11	0,20	0,16
***Inhibitory***	38	30	30	34	26	20
*Complete*	27	25	16	18	13	7
*Incomplete*	38	30	30	16	13	14
*Total surface area (complete only)*	61,49	43,26	22,10	42,84	23,67	17,87
*Mean for each bouton*	2,28	1,73	1,38	2,38	1,82	2,55
*Standard deviation*	1,42	0,89	0,57	1,46	0,94	1,82
*Total volume (complete only)*	6,76	3,99	1,70	4,13	1,95	1,14
*Average*	0,25	0,16	0,11	0,23	0,15	0,19
*Standard deviation*	0,30	0,13	0,07	0,19	0,10	0,11
***Unknown***	49	95	23	38	74	52
*Complete*	30	48	15	22	29	16
*Incomplete*	49	95	23	16	45	36
*Total surface area (complete only)*	30,44	59,72	15,68	17,94	32,79	14,14
*Mean for each bouton*	1,01	1,24	1,05	0,82	1,13	0,88
*Standard deviation*	0,54	0,90	0,46	0,42	0,65	0,44
*Total volume (complete only)*	1,91	4,49	1,01	1,05	2,17	0,80
*Average*	0,06	0,09	0,07	0,05	0,07	0,05
*Standard deviation*	0,05	0,11	0,04	0,03	0,06	0,03
**Spines**			** **			
***Total***	124	152	180	158	128	171
*Complete*	74	108	122	105	85	107
*Incomplete*	49	44	58	53	41	64
*Total surface area (complete only)*	65,47	98,98	89,52	82,52	92,98	78,35
*Mean for each spine*	0,88	0,92	0,73	0,79	1,09	0,73
*Standard deviation*	0,70	0,72	0,56	0,66	0,79	0,67
*Total volume (complete only)*	5,15	7,68	6,48	6,17	7,89	5,74
*Mean for each spine*	0,07	0,07	0,05	0,06	0,09	0,05
*Standard deviation*	0,08	0,08	0,06	0,07	0,09	0,07
*Total Lenght*	90,96	139,31	135,94	122,16	95,62	127,45
*Mean for each spine*	1,60	1,33	1,39	1,44	1,47	1,45
*Standard deviation*	0,86	0,54	0,74	0,69	0,55	0,61
**Synapses**	** **	** **	** **	** **	** **	** **
***Total***	189	167	211	212	185	171
*Complete*	159	129	146	163	135	126
*Incomplete*	30	38	65	49	50	45
*Total surface area (complete only)*	23,39	25,00	17,02	24,56	22,79	20,11
*Mean for each synapse*	0,15	0,19	0,12	0,15	0,17	0,16
*Standard deviation*	0,13	0,15	0,10	0,17	0,14	0,15
***Excitatory***	131	109	165	138	122	128
*Complete*	104	91	119	108	91	94
*Incomplete*	27	18	46	30	31	34
*Total surface area (complete only)*	15,73	18,76	14,26	17,67	17,60	16,71
*Mean for each synapse*	0,15	0,21	0,12	0,16	0,19	0,18
*Standard deviation*	0,14	0,15	0,10	0,15	0,16	0,17
***Inhibitory***	34	25	30	29	32	23
*Complete*	33	21	16	23	22	20
*Incomplete*	1	4	14	6	10	3
*Total surface area (complete only)*	5,59	3,98	1,85	1,78	3,11	2,66
*Mean for each synapse*	0,17	0,19	0,12	0,08	0,14	0,13
*Standard deviation*	0,12	0,11	0,09	0,06	0,09	0,11
***Unknown***	24	33	16	45	31	20
*Complete*	22	17	11	32	22	12
*Incomplete*	2	16	5	13	9	8
*Total surface area (complete only)*	2,07	2,26	0,91	5,10	2,08	0,74
*Mean for each synapse*	0,09	0,13	0,08	0,16	0,09	0,06
*Standard deviation*	0,08	0,18	0,07	0,24	0,06	0,04
**Mitochondria**	** **	** **	** **	** **	** **	** **
***Total***	187	181	202	219	210	183
*Axonal*	130	130	146	149	149	137
*Dendritic*	43	41	39	54	53	41
*Undefined*	14	10	17	15	8	5

Total number and averaged measurements of length, surface area and volumes on reconstructed objects from the 6 volumes of neuropil.

The 3D reconstructions from each volume were exported as object files (.obj) from either FIJI or Ilastik and imported into the Blender software for visualization. Morphological analysis was performed within this program using the NeuroMorph Measurement Tools [33] (https://neuromorph.epfl.ch; Blender version 2.9, www.blender.org). Each object is represented by a surface mesh comprising vertices, edges, and faces which can be fully annotated using this software. The complete set of images for each of these subvolumes and the corresponding reconstructions are provided here: http://datadryad.org/review?doi=doi:10.5061/dryad.bh78sn5

Within each of one of these image stacks, we quantified the volume, surface area, and length of all the axons and their corresponding boutons, as well as dendrites and their spine heads. The surface area of each asymmetric synapse was measured and each of these objects was then assigned an identifier tying it to its presynaptic bouton and postsynaptic spine, or dendrite (details given in Table 2). All the data were exported as a single data sheet, and all statistical analyses were then performed using Prism 6.0 (GraphPad). To measure the 3D arrangement of vesicles in a single bouton we again used NeuroMorph and the Synapse Vesicle Distances tool. Within the center of each vesicle, a sphere was placed which approximately filled the structure. This made it hard to double count vesicles. The center of each of these spheres was used to calculate the Euclidian distance between the vesicle and the nearest point on the mesh model representing the presynaptic membrane. These values were then separated into bins at 30 nm intervals.

## Results

### Reduced cortical thickness in aged animals

Coronal sections at the level of the somatosensory cortex (**Fig 1A**) showed a 15.6% reduction in the total cortical thickness in the aged animals (**Fig 1B**; adult, 1.03 ± 0.047 mm; aged, 0.87 ± 0.003 mm; p = 0.029, unpaired t-test). Measurements of layer 1 also showed a reduction of 19.4% (adult, 0.103 ± 0.004 mm; aged, 0.083 ± 0.002 mm; p = 0.0009, unpaired t-test). These measurements were made at the level of the larger barrels in layer IV of the somatosensory cortex. The Nissl staining showed that cell bodies, myelin, and the boundaries between the different layers had a similar appearance in both groups. Other than the difference in thickness, the histological sections were indistinguishable between the aged and adult animals.

Although we are not able to estimate the numbers of cells in this brain region from the single sections, as we made no estimate of the size of this cortical area, or use a stereological counting method, we were nevertheless interested in comparing the difference in cell densities in the sections used to measure the cortical thickness. The density of cells, measured in bins at different depths from the pial surface was the same (**Fig 1D**; Kolmogorov-Smirnov test, p > 0.9).

### Synaptic density comparison between adult and aged animals

The density of synapses in layer 1 was measured from six FIBSEM image stacks. These ranged in size from 663 to 1417 cubic micrometers in each mouse (**Fig 2**). The total synaptic density was 16.5% lower than in adults (**Fig 2A**; 1.40 ± 0.006 per μm^3^ for adults; 1.17 ± 0.013 per μm^3^ for aged; p < 0.001, unpaired t-test). This decrease is caused by a loss of asymmetric and symmetric synapses (presumed excitatory and inhibitory, respectively) on dendritic spines (**Fig 2B, 2C** and **2D**; asymmetric, 1.05 ± 0.03 per μm^3^ for adults; 0.96 ± 0.02 per μm^3^ for aged; p < 0.001, unpaired t-test; symmetric, 0.07 ± 0.01 per μm^3^ for adults; 0.02 ± 0.006 per μm^3^ for aged; p = 0.0028, unpaired t-test), and on the dendritic shaft. However, fewer synapses of both types are found on shafts, and the difference between aged and adult densities did not reach significance (**Fig 2C** and **2D**; asymmetric 0.17 ± 0.03 per μm^3^ adults; 0.11 ± 0.02 per μm^3^ aged; t-test, p = 0.09; symmetric 0.08 ± 0.007 per μm^3^ adult; 0.065 ± 0.003 per μm^3^ aged, p = 0.27).

### Inhibition—excitation changes

The percentage decrease in synapse density is different between the excitatory and inhibitory types. While there is a 12.5% reduction in total asymmetric synapses (1.23 ± 0.018 per μm^3^ for adults; 1.07 ± 0.03 per μm^3^ for aged; p < 0.01, unpaired t-test), the symmetric type is reduced by almost half (45%; 0.15 ± 0.009 per μm^3^ for adults; 0.08 ± 0.002 per μm^3^ for aged; p = 0.013, unpaired t-test). On the dendritic spines there is a 9.3% reduction of the asymmetric type, but a 74.7% reduction in the symmetric (**Fig 2C** and **2D**). This results in a 36.6% decrease of the inhibitory to excitatory ratio in the aged group (0.123, adults; 0.078, aged).

### Density of multi-synaptic boutons (MSBs)

The number of boutons with more than one asymmetric synapse (multi-synaptic, MSB) was also counted. This was only possible for the asymmetric (excitatory) synapses. Clearly defined boutons, with symmetric synapses, were often ill-defined, and it was difficult to unambiguously define the start and end of the varicosity. The density of multi-synaptic boutons, synapsing on spines, dendrites, or on both, was higher in adults, compared to aged mice, but did not reach statistical significance (MSBs on spines: 0.078 ± 0.02 per μm^3^ adults; 0.065 ± 0.01 per μm^3^ aged; t-test, p = 0.61; MSBs on dendrites: 0.0095 ± 0.004 per μm^3^ adults; 0.0013 ± 0.001 per μm^3^ aged; t-test, p = 0.09; MSBs on spines and dendrites: 0.025 ± 0.014 per μm^3^ adults; 0.0045 ± 0.031 per μm^3^ aged; t-test, p = 0.21).

### Synaptic size

While identifying each synapse in the serial EM images, to measure their densities, an estimate was also made of their size. This was done by placing a circle whose diameter matched the length of the synaptic contact on the image where it appeared largest (Fig 3**A**). To verify that these measurements corresponded to the synapse size, we took 57 synapses that had also been reconstructed in 3D (Fig 3**B**). There was a close correlation between these maximum diameter measurements and their exact surface area measured from the 3D reconstructions, giving an R^2^ value of 0.84 (**Fig 3C**). The synapse size measurements showed that aged animals had synapses that were 11.6% larger (**Fig 3D** *inset*; 377.8 ± 3.2 nm for adults; 427.7 ± 3.9 nm for aged; p<0.0001, Kolmogorov-Smirnov test). A size frequency distribution graph (**Fig 3D**) shows a skew to the right suggesting that both populations can be approximated by a log normal function (**Fig 3E** and **3F**; adult, red fit curve R^2^ = 0.96; aged, blue fit curve, R^2^ = 0.94).

To compare the shapes of synapses we used a machine learning approach for detecting synapses [34] (**Fig 3G**). This automated segmentation algorithm uses image features that take spatial context into account. We found this to be a reliable way of obtaining a reconstruction of the contact site. From the complete stacks, used to estimate synaptic densities, we saw a significant increase in the percentage of perforated synapses (**Fig 3H**: mean of 3.81%, adult; 8.1%, aged: p = 0.014 unpaired t-test). We only considered synapses as being perforated if there was a clear hole. We did not include in our classification those with a complex, or horse-shoe, shape as being perforated.

### Synapses per unit length

The tissue used in this analysis was preserved using a chemical fixation protocol, similar to other EM studies making estimates of synapse density. Chemical fixation is known to cause tissue shrinkage [35], and we cannot rule out that this is different at the two ages. We, therefore, refined our measurements further by making dense reconstructions of all the cellular elements in 5 x 5 x 5 μm cubes from within the larger stacks used for the synapse counts (**Fig 4B**). Similar to the larger stacks, these sub-volumes showed an 11.8% increase synapse surface area in the aged animals (**Fig 4C**; 0.15 ± 0.007 μm^2^ for adults; 0.17 ± 0.008 μm^2^ for aged; p = 0.04, Kolmogorov-Smirnov test). These also contained significantly more axon, a 20.7% increase per unit volume (**Fig 4D**; 8.15 ± 0.19 μm of axons per μm^3^ for adults; 10.27 ± 0.88 μm of axons per μm^3^ for aged; p = 0.016, unpaired t-test). The effect on the dendrite, however, was less prominent and did not reach statistical significance (**Fig 4D**; 1.37 ± 0.06 μm of dendrites per μm^3^ for adults; 1.53 ± 0.12 μm of dendrites per μm^3^ for aged; p = 0.3, unpaired t-test). This analysis was not able to classify all axons according to whether they made a symmetric or asymmetric synapse as many made no synapses within the sub-volume, or at any other point in the larger stack. Taking all synapses together, there is one synapse every 5.82 μm of axon in the adult animals and every 8.62 μm in the aged ones. In view of the lower synapse density in the older animals, and higher axonal content, the aged animals have a 33% decrease in the number of synapses per unit length of axon (**Fig 4E**; 0.173 ± 0.005 per μm of axons for adults; 0.116 ± 0.011 per μm of axons for aged; p = 0.008, unpaired t-test), but also a 25% decrease in synapses per unit length of dendrite (adult, 1.03 ± 0.046 synapses per μm of dendrite; aged, 0.776 ± 0.072 synapses per μm of dendrite; t-test, p = 0.04).

The reduction in the synapse density in the aged animals appears to be balanced by an increase in their size. Total synaptic surface, per unit volume of neuropil, or per unit axon or dendrite length, is indeed not significantly different between the two groups (**Fig 4F** and **4G**).

### Vesicle analysis

**Fig 5:**
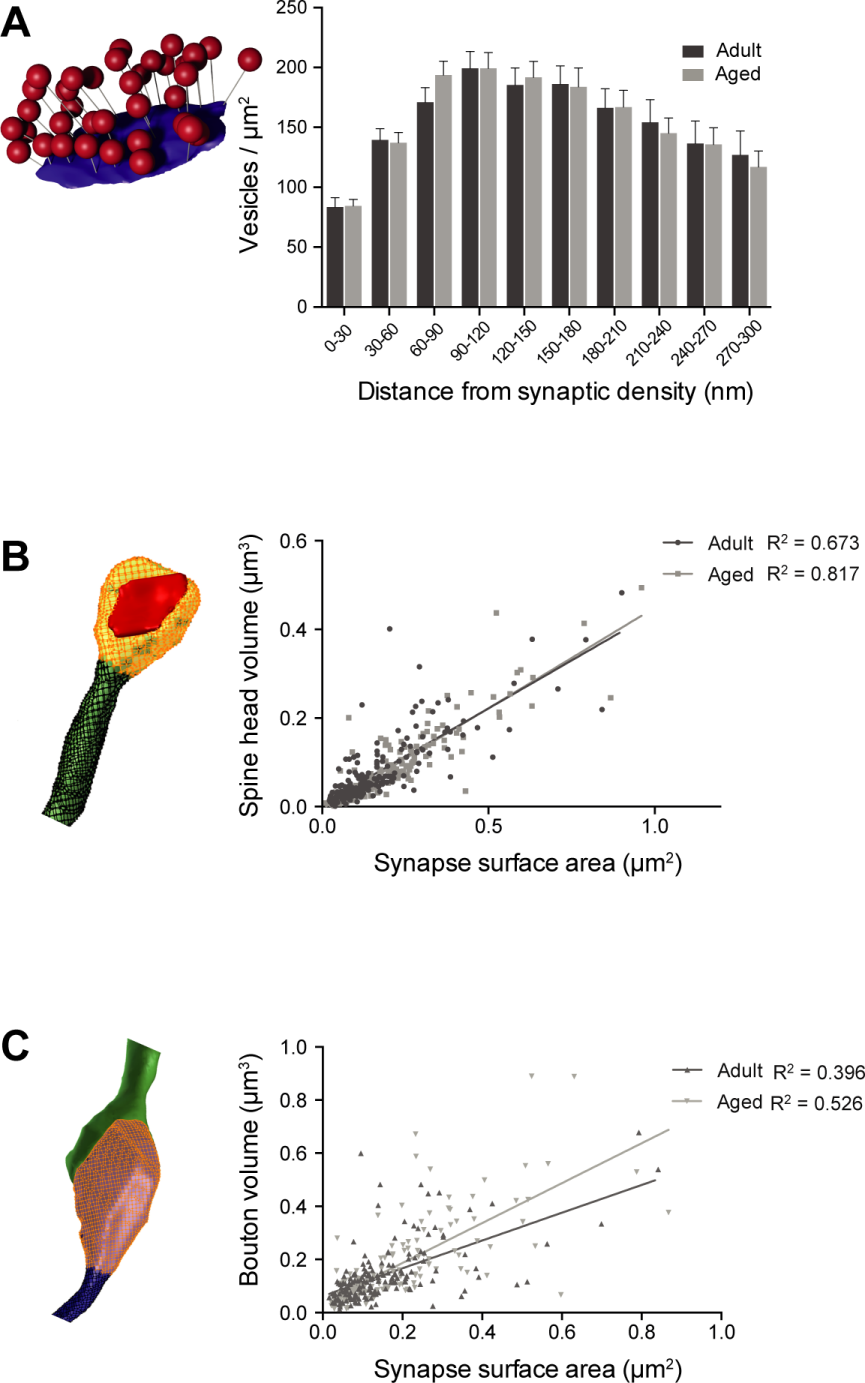
Spine and bouton volume correlate closely with synapse size in the aged and adult neuropil. **A**, Histogram showing the distribution of vesicle density normalized to the synaptic contact surface area shows no difference between aged and adult animals (p = 0.4, Kolmogorov-Smirnov test). **B**, Correlation between spine head volume and synaptic surface area for adult (N = 207; R^2^ = 0.673) and aged (N = 197; R^2^ = 0.817) mice. The slopes are not significantly different (ANOVA, p = 0.42). **C**, Correlation between volume of excitatory boutons and synaptic surface area for adult (N = 169; R^2^ = 0.369) and aged (N = 146; R^2^ = 0.526) mice. The slopes are significantly different (ANOVA, p = 0.0045).

Do the changes in synapse number and size in aged neuropil disrupt the correlations between parameters such as synapse size, vesicle density, vesicle docking, spine size, and bouton size [36, 37]? To address these points, we first plotted the position of all vesicles, using the NeuroMorph 3D Drawing tool, contained in single synaptic boutons synapsing with dendritic spines (38 in adult, and 41 aged). The pre-synaptic density was also mapped in 3D. In total, 9652 vesicles were plotted in the adult boutons and 8739 in aged ones. The vesicle density within the boutons was the same between the two groups (1847 ± 156.7 vesicles per μm^3^ for adults; 2225 ± 243.5 vesicles per μm^3^ for aged; p = 0.2, unpaired t-test). We also measured the distance of each vesicle to the pre-synaptic membrane and grouped them into bins at 30 nm distance intervals. This showed that at both ages the density of vesicles around the synapse was the same (**Fig 5A**; p = 0.4, Kolmogorov-Smirnov test).

To investigate the correlations between synapse size and spine and bouton volume we measured every bouton, spine head, and area of the connecting synapse within the sub-volumes. We did not distinguish between *en passant* boutons and *boutons terminaux*.

As in other studies, we found at both ages high correlation between the size of the synapse, and the volume of the spine head [9, 10, 38, 39, 11] (**Fig 5B**; adults, n = 207, r = 0.82, p < 0.0001, linear fit; aged, n = 197, r = 0.9, p < 0.0001, linear fit). And similarly for the size of the synapse and volume of single synapse boutons (**Fig 5C**; adults, n = 169, r = 0.6, p < 0.0001; aged, n = 146, r = 0.72, p < 0.0001). We did not include the MSBs in this analysis.

In view of the larger synapses in the aged animals, the strong correlation between synapse size and spine volume would suggest this group to have larger spines. We tested this, using all the complete spines contained within the sub-volumes. However, we found no difference between the two groups (0.075 ± 0.005 μm^3^ for adults, N = 220; 0.079 ± 0.006 μm^3^ for aged, N = 216; p = 0.6, unpaired t-test). The average size of the spines was similar to those measured in layer 4 of the same region of young adult mice [40] (60–65 days, 0.06 ± 0.04 μm^3^). We could also not detect a difference in bouton sizes between the two groups (0.189 ± 0.01 μm^3^ for adults, N = 244; 0.195 ± 0.01 μm^3^ for aged, N = 189; p = 0.67, unpaired t-test). The average bouton size is larger than the non-thalamocortical type measured in mouse layer 4 [11], but smaller than the thalamocortical ones [11, 39]. We do not know the identity of axons in our samples, but this difference is probably a reflection of very different bouton population than those measured in the deeper layer.

### Mitochondrial content

Our analyses show that the neuropil in the aged mice maintains strong correlations between spine, bouton and synapse size, as well as unchanged vesicle distributions, and total synaptic surface area. This suggests compensatory mechanisms to maintain total synaptic currents, despite a drop in synapse number.

**Fig 6:**
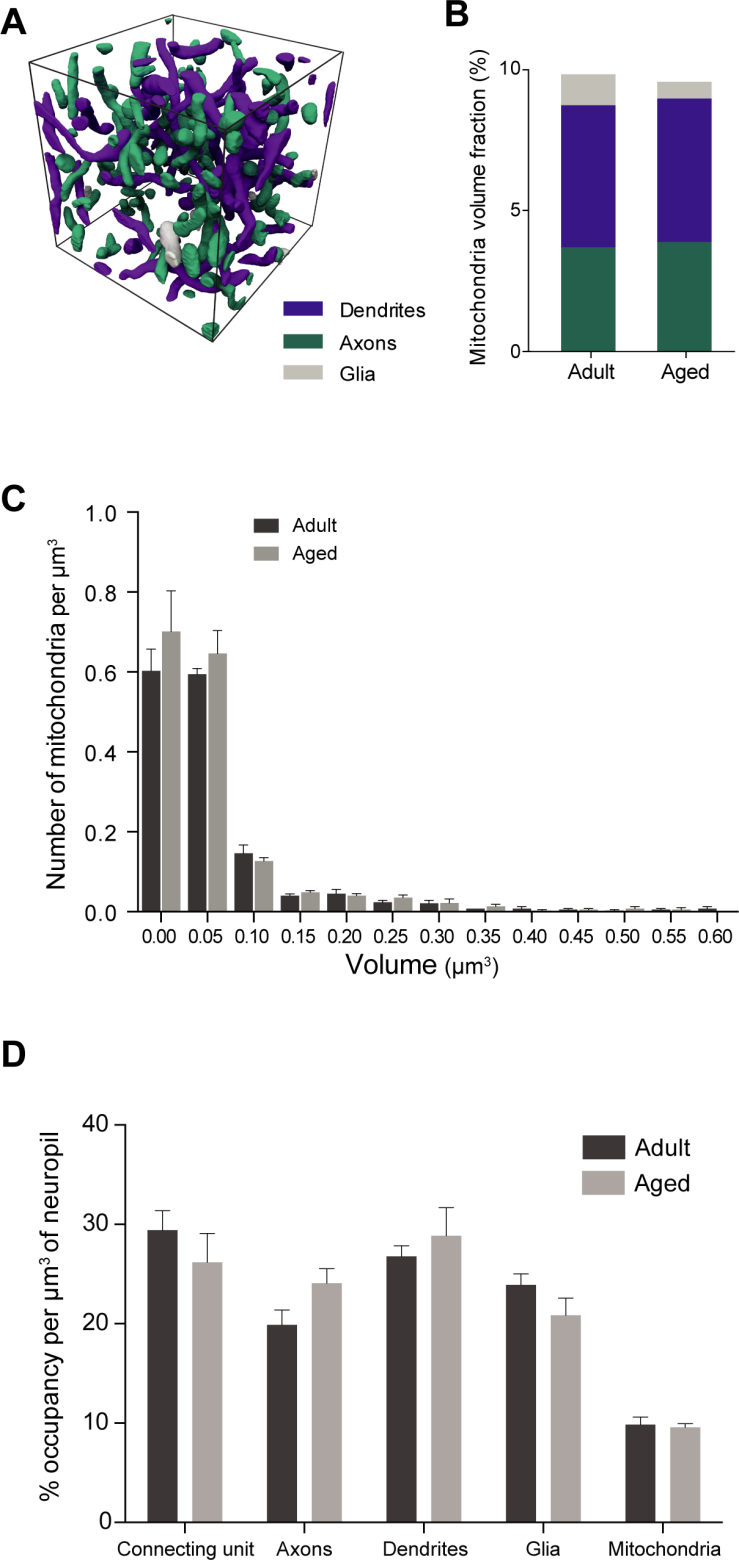
Different compartments unchanged in aged neuropil. **A**, The volumes of every mitochondria were measured from reconstructions in the sub-volumes. **B**, The volume fraction of mitochondria in axons, dendrites and glia did not change between the two groups (total; adults 9.8 ± 0.76%, aged 9.5 ± 0.37%, p = 0.76; axons, adults 3.7 ± 0.15%, aged 3.9 ± 0.45%, p = 0.71; dendrites, adults 5.1 ± 0.7%, aged 5.1 ± 0.4%, p = 0.94; others, adults 1.1 ± 0.25%, aged 0.6 ± 0.26%, p = 0.22; unpaired t-test). **C**, The distribution of mitochondrial sizes is not different between the adult and aged mice. Frequency distribution histogram shows the densities of different mitochondria, grouped by volume (adults 0.116 ± 0.06 mitochondria per μm^3^, aged 0.127 ± 0.07 mitochondria per μm^3^, p = 0.99; Kolmogorov-Smirnov test). **D**, Percentage of occupancy of connecting units (boutons and spines combined), axons, dendrites, glia, as well as mitochondria, per cubic micrometer of neuropil from the each of 6 sub-volumes. None of the differences are statistically significant (Connecting unit, adults 29.4 ± 1.96%, aged 29.4 ± 1.96%, p = 0.4; axons; adults, 19.9 ± 1.49%, aged 24.1 ± 1.46%, p = 0.11; dendrites, adults 26.7 ± 1.09%, aged 28.9 ± 2.81%, p = 0.52; glia, adults 23.9 ± 1.1%, aged 20.8 ± 1.73%, p = 0.21; mitochondria, adults 9.8 ± 0.76%, aged 9.5 ± 0.37%, p = 0.76; N = 3 per each group, unpaired t-test).

Mitochondrial shape has been implicated in altered synapse function [40]. We, therefore, reconstructed all the mitochondria in these sub-volumes, classifying them according to whether they were present in dendrites, or axons (**Fig 6**). Measurements of the total mitochondria volumes showed no difference between the two ages (**Fig 6B**; adults 9.8 ± 0.76%, aged 9.5 ± 0.37%, p = 0.76; unpaired t-test). Differences in the size distribution of the mitochondria (**Fig 6C**; adults 0.116 ± 0.06 mitochondria per μm^3^, aged 0.127 ± 0.07 mitochondria per μm^3^, p = 0.99; Kolmogorov-Smirnov test), might indicate an influence on possible fission and fusion mechanisms. Although this shows no difference between the two groups, it should be pointed out that mitochondria in the axons and dendrites have very different lengths. Those in the axons are typically short and confined to regions of synaptic content (i.e. boutons), whereas those in the dendrites can stretch tens of micrometers and would not, therefore, be fully contained in our sub-volumes. As irregular shaped mitochondria, and in particular those with donut-shapes, have been associated with a decline in working memory, and a reduction in total docked vesicle number in aged monkey prefrontal cortex [41], we counted these in each of the volumes. We found only one in all the adult volumes (1 out of 570; 0.18%), and eight in the aged (8 out of 611; 1.31%). All of these were present in excitatory axonal boutons, and none in dendrites.

## Discussion

This study shows, through the analysis of dense reconstructions, how the structure of the neuropil is altered through aging. The method gives an unbiased view of the elements associated with the connectivity, which is particularly relevant when counting features within a volume that are susceptible to shrinkage through chemical fixation [34]. It shows, like other studies, a synaptic loss, but in addition, a compensatory increase in excitatory synapse size to keep the same amount of synaptic surface per unit volume of neuropil, or per unit length of neurite. There is also an increase in the ratio between excitation and inhibition, and no indication of excitatory synaptic functional loss as the correlations between synapse, spine, and bouton sizes, are maintained.

Our measurements of synaptic density, normalized with volume as well as neurite length, are an important verification that there is a true loss of synaptic connections along the neurites. The total length of axon measured in the adult volumes was remarkably similar to a previous estimate in mouse cortex (5.69 micrometers of axon per synapse, [42] compared to 5.78 measured here). An estimate in the rat hippocampus was 4–5 micrometers per synapse [43]. In aged neuropil the value was considerably higher (8.62). This is partly due to the increase in axonal length per unit volume, which is difficult to reconcile. An increased amount of axon in aged neuropil seems counter-intuitive, or at least we are not aware of any information that aging causing an increase in axonal growth, but rather the contrary, with evidence of branch loss [3, 21]. This could merely be a sampling issue, where the volume fraction has increased slightly due to the reduction of other elements, though we could not detect this in our volume reconstructions (**Fig 6D**). A combination of factors is more likely. Dendritic regression occurs with aging in different brain areas [3, 44, 45], although, this has not been shown in the whisker barrel cortex [46], but there is also a reduction in the number of spines, as well as fewer axonal boutons. These changes would result in more space in which axons could be found if their individual sizes do not change. We also cannot rule out a change in the population of axons present, with a greater number of thinner ones. A significant reduction in the number of inhibitory synapses could be an indication also of a change in the proportion of certain axonal classes.

The largest decrease in inhibition is seen on spines. As spines with an inhibitory input also receive an excitatory one, and from boutons that are thought to be of thalamic origin, this would suggest a reduction in the GABAergic innervation that regulates sensory information arriving in this cortical layer [47, 48]. The neurons responsible for this inhibition could be the somatostatin-expressing Martinotti cells whose axons branch extensively in the upper layers of the cortex [49, 50], but without any specific labelling we can only speculate.

A change in excitatory and inhibitory balance has been seen previously in the aged auditory cortex of the rat and measured with electrophysiological recordings [23]. This is also supported by immunocytochemistry with a reduction in GABA related enzymes in the aging rodent sensory cortex [37, 51, 52]. We wonder whether the reduction in the inhibition is as a result of reduced sensory-induced activation of the cortex. An increase in inhibition of dendritic spines is seen in layer IV of the somatosensory cortex after over-stimulation of peripheral receptors by magnetically twitching single whiskers. The functional consequence is a reduction in response properties of neurons in the region of the cortex corresponding to the stimulated whisker [53]. In less active, aged animals could this cause the opposite effect, with a reduced inhibition to dendritic spines? Rats deprived of whisker information to the cortex from an early age exhibit significant reduction in the number of inhibitory synapses on spines, as do rats with monocular deprivation [54].

As well as reduction of inhibition we found an increase in synapse size. Light microscopy analyses of cortical neurons have implied increased contact size with dendrites showing reduced numbers of thinner spines [19, 35]. This is in contrast to EM analysis of hippocampal synapses that show a decrease in synapse size [55]. We also show an increase in the number of perforations, which has functional implications. Larger, perforated synapses with more AMPA receptors [56, 57] elicit larger EPSPs. Increased numbers of perforated synapses are found in hippocampal tissue after giving potentiating stimuli. Smaller, non-perforated ones represent the silent, more plastic and younger connections [58, 10, 59]. We also measure an even tighter correlation in the aged animals between the spine volume and synapse size, reflecting a reduction in the variability in spine size and a loss in the number of thinner spines, i.e. those considered to be more transient [60, 61]. This also fits with immunocytochemistry and functional analyses of aged mammalian cortices showing decreased expression of NMDA receptors [62, 63, 64], and a larger non-NMDA response than younger animals [37].

These comparisons of layer 1 neuropil morphology indicate a maturation of connectivity, with few smaller connections, suggesting a neuropil with fewer transient connections [57, 59], fewer structures that may serve as silent synapses [65] and with less potential for the storage of new information [66]. Our analysis, however, is only structural, and although there is consensus on parameters such as synapse size correlating with receptor content [67], we can only assume this also holds for the aged brain. Age-related changes to the sensitivity of some receptor subunits [68] could mean that synapses only appear larger in the electron micrographs, and compensatory mechanisms have inserted greater numbers of receptors to maintain the synapse function. Although we were not able to test this directly, we also measured the numbers of vesicles at the pre-synaptic membrane and found the same numbers at both ages. Since synapse size correlates with the numbers of docked vesicles [36], if larger synapses in the aged brain do not reflect an increased insertion of fully functional receptors, then we may not expect the same densities of vesicles per unit area of contact site. Or at least it seems counterintuitive that synapses with larger numbers of less functional receptors are complemented with a greater number of docked vesicles per unit area. This indirectly points towards synapses in adult and aged brains displaying identical synaptic architecture. This increase in synapse size, however, has functional implications as larger synapses show a greater release probability [69], therefore improving the reliability of neurotransmitter release and a tighter tuning of the excitatory connections.

This change in the contact size could compensate for their reduced numbers. We find the same amount of synapse surface area, per unit volume, as well as per unit axon and dendrite length. Could this be an indication that whatever differences in connectivity may occur in aging, due to the pruning of the axonal and dendritic branches, compensatory mechanisms at the local level maintain the levels of synaptic connectivity? A comprehensive structural analysis of pyramidal neurons in aged and adult monkey showed that despite significant changes to dendrites and spine numbers their fundamental electrophysiological properties had not differed [14]. Therefore, the fixed amount of synaptic surface area per unit volume, and per unit length of neurite, that we find here could also be an indication of metabolic limits imposed by the tissue. As we found the same amount of mitochondria at both ages, this raises the possibility that the total mitochondrial volume, providing the energy, plays an important role in determining how much synaptic machinery can be maintained, within the neuropil.

Therefore, during aging the extent of a neuron’s axonal and dendritic reach may be reduced due to a decrease in its ability to maintain all the necessary biochemical processes across long distances, and this results in a reduction of synaptic contacts, However, the plasticity of the remaining connectivity compensates to help maintain the excitability of the neurons, but only as much as the energy supply can provide.

## Supporting information

S1 Raw Data File File showing data used in all figures. (XLSX)
